# Decoding Diffusivity in Multiple Sclerosis: Analysis of Optic Radiation Lesional and Non-Lesional White Matter

**DOI:** 10.1371/journal.pone.0122114

**Published:** 2015-03-25

**Authors:** Alexander Klistorner, Nikitha Vootakuru, Chenyu Wang, Con Yiannikas, Stuart L. Graham, John Parratt, Raymond Garrick, Netta Levin, Lynette Masters, Jim Lagopoulos, Michael H. Barnett

**Affiliations:** 1 Department of Ophthalmology, Save Sight Institute, University of Sydney, Sydney, Australia; 2 Australian School of Advanced Medicine, Macquarie University, Sydney, NSW, Australia; 3 Westmead Hospital, Sydney, NSW, Australia; 4 Brain and Mind Research Institute, University of Sydney, Sydney, NSW, Australia; 5 North Shore Hospital, Sydney, NSW, Australia; 6 St. Vincent Hospital, Sydney, NSW, Australia; 7 Hadassah Hebrew University Medical Center, Jerusalem, Israel; Institute Biomedical Research August Pi Sunyer (IDIBAPS) - Hospital Clinic of Barcelona, SPAIN

## Abstract

**Objectives:**

Diffusion tensor imaging (DTI) has been suggested as a new promising tool in MS that may provide greater pathological specificity than conventional MRI, helping, therefore, to elucidate disease pathogenesis and monitor therapeutic efficacy. However, the pathological substrates that underpin alterations in brain tissue diffusivity are not yet fully delineated. Tract-specific DTI analysis has previously been proposed in an attempt to alleviate this problem. Here, we extended this approach by segmenting a single tract into areas bound by seemingly similar pathological processes, which may better delineate the potential association between DTI metrics and underlying tissue damage.

**Method:**

Several compartments were segmented in optic radiation (OR) of 50 relapsing-remitting MS patients including T2 lesions, proximal and distal parts of fibers transected by lesion and fibers with no discernable pathology throughout the entire length of the OR.

**Results:**

Asymmetry analysis between lesional and non-lesional fibers demonstrated a marked increase in Radial Diffusivity (RD), which was topographically limited to focal T2 lesions and potentially relates to the lesional myelin loss. A relative elevation of Axial Diffusivity (AD) in the distal part of the lesional fibers was observed in a distribution consistent with Wallerian degeneration, while diffusivity in the proximal portion of transected axons remained normal. A moderate, but significant elevation of RD in OR non-lesional fibers was strongly associated with the global (but not local) T2 lesion burden and is probably related to microscopic demyelination undetected by conventional MRI.

**Conclusion:**

This study highlights the utility of the compartmentalization approach in elucidating the pathological substrates of diffusivity and demonstrates the presence of tissue-specific patterns of altered diffusivity in MS, providing further evidence that DTI is a sensitive marker of tissue damage in both lesions and NAWM. Our results suggest that, at least within the OR, parallel and perpendicular diffusivities are affected by tissue restructuring related to distinct pathological processes.

## Introduction

Multiple sclerosis (MS) is a complex disease of the CNS, characterized by inflammation, demyelination, neuro-axonal loss and gliosis. While conventional MRI plays a crucial role in the diagnosis of MS, its contribution to understanding mechanisms that underpin the disease and the relationship to pathological features is limited due to low specificity.

Significant expansion of the MS therapeutic armamentarium over the last five years has re-emphasized the critical need for reliable *in vivo* markers of neurodegeneration and de/remyelination.

Diffusion tensor imaging (DTI) is sensitive to the microstructural organisation of white matter tracts and has been suggested as a new promising tool that provides greater pathological specificity than conventional MRI, helping, therefore, to elucidate disease pathogenesis and monitor therapeutic efficacy [1].

However, the pathological substrates that underpin alterations in brain diffusivity are yet to be fully delineated. It was suggested that diffusivity perpendicular to the white matter fiber tracts (Radial Diffusivity, RD) is restricted by both the axonal membrane and the myelin sheath. While RD is considerably lower than diffusivity parallel to the fibers (Axial Diffusivity, AD), resulting in high fractional anisotropy (FA), demyelination can significantly “modulate” this relationship [2]. This has prompted speculation that RD may potentially be used as a marker of myelination [3][4][5]. Surprisingly, recent studies have failed to demonstrate an unequivocal relationship between increased RD and the degree of demyelination, suggesting that this measure is not pathologically specific [6]. Similarly, a strong association between AD and axonal pathology, described in earlier animal models [7] has not been corroborated in a recent post-mortem study [8]. This is not entirely surprising given that post-mortem and animal studies may not be directly comparable or applicable to *in vivo* human pathology. On the other hand, clinical studies of diffusivity are difficult to validate since histological correlations are not feasible and proof of specificity of the diffusion measure in question can only be indirectly deduced.

The elucidation of mechanisms underlying altered diffusivity is hampered by potential misalignment between eigenvectors and corresponding underlying tissue structures [9]. This confounding effect has been partly abrogated by the recent introduction of tract-specific techniques, which provide high fiber coherency.[8][10][11].

In the current study we sought to extend this approach by segmenting the single tract into areas (compartments) bound by seemingly similar pathological processes. We hypothesized that this may better delineate the potential association between the DTI metrics and underlying tissue damage. As such, the current study represents the first attempt to analyse diffusivity changes within a single white matter tract using tissue compartmentalization approach.

## Materials and Methods

Study was approved by Sydney University Ethics Committee. All procedures followed the tenets of the Declaration of Helsinki and written informed consent was obtained from all participants

### Subjects

Fifty consecutive Relapsing-Remitting MS (RRMS) patients without a history of clinical optic neuritis (ON) in at least one eye were enrolled. RRMS was defined according to standard criteria [12]. History of ON was determined based patient’s clinical notes. Patients with any other systemic or ocular disease were excluded. Fifteen normal age- and sex-matched controls were also examined.

### MRI protocol

The following sequences were acquired using a 3T GE Discovery MR750 scanner (GE Medical Systems, Milwaukee, WI):

- Pre- and post contrast (gadolinium) Sagittal 3D T1: GE BRAVO sequence, FOV 256mm, Slice thickness 1mm, TE 2.7ms, TR 7.2ms, Flip angle 12°, Pixel spacing 1mm. Acquisition Matrix (Freq.× Phase) is 256×256, which results in 1mm isotropic acquisition voxel size. The reconstruction matrix is 256x256.
- FLAIR CUBE; GE CUBE T2 FLAIR sequence, FOV 240mm, Slice thickness 1.2mm, Acquisition Matrix (Freq.× Phase) 256×244, TE 163ms, TR 8000ms, Flip angle 90°, Pixel spacing 0.47 mm. The reconstruction matrix is 512x512.
- Whole brain diffusion-weighted images using a spin echo, 64 directions, FOV 256 mm, Acquisition Matrix (Freq.× Phase) 128×128, slice thickness 2mm, TE 83ms, TR 8325ms, b-value = 1000 and number of b = 0 acquisitions = 2. The reconstruction matrix is 256x256.

### Tractography

Probabilistic tractography was used to reconstruct OR fibers as previously described in detail [13]. Briefly, after eddy-current correction and motion compensation, DTI and FLAIR T2 images were co-registered to the high resolution T1 structural image. Implementation of probabilistic tractography requires the presense of both ROIs, which, in case of optic radiation, are LGN and occipital cortex. To identify LGN, which is practically invisible on structural images, optic tract fibers were followed from chiasm using deterministic tractography (10 mm ROI placed on chiasm was used for seeding of deterministic algorithm). Position of LGN was determined based on termination of optic tract fibers, at which point circular ROI (diameter 7 mm) was placed.

An occipital cortex ROI for probabilistic tractography of the OR covering the calcarine sulcus was drawn manually in each hemisphere using the high resolution T1 structural image. Procedure was performed using editable ROI function of MrDiffusion software (http://sirl.stanford.edu/software/). Probabilistic tractography (ConTrack part of MrDiffusion package) was then employed between the LGN and calcarine ROIs. Parameters for probabilistic tractography described by Sherbondy et al [14]. Initially 70000 fibers were collected for OR tractography, of which the 30000 best fibers were selected by a scoring algorithm. OR fibers were then manually cleaned using Quench software (http://sirl.stanford.edu/software/). Mayer loop was clearly visible in all OR reconstructions.

### Lesion identification

MS lesions were identified on the co-registered T2 FLAIR images and segmented automatically using ITK-SNAP 2 (http://www.itksnap.org). We took extreme care to ensure that co-registration was accurate-each image was visually examined by two authors (AK and CW) and agreement on accurate co-registration was reached in every case. Lesions were then intersected with OR fibers to identify and measure the volume of T2 FLAIR lesions within and outside of the OR.

### OR selection

**Fig 1:**
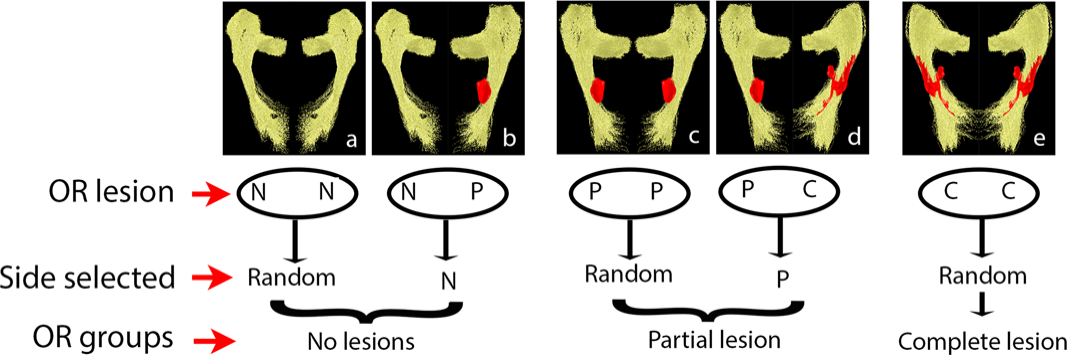
Selection of the Optic Radiation. Following criteria has been used for selection of OR. For non-lesional group:(a) in patients with no (N) OR lesions on both sides, OR was selected randomly, (b) in patients with no OR lesions on one side and partial (P) OR lesion on the other, OR with no (N) lesions was selected. For partial lesion group: (c) in patients with lesions that partially involve the OR in both hemispheres, OR was selected randomly, (d) in patients with partial lesion on one side and complete (C) lesion-on the other, OR with partial (P) lesion was selected. For complete lesion group: (e) in patients with lesions occupying the entire cross-section of the OR in both hemispheres OR was selected randomly. No patients had no lesion on one side and complete lesions on the other side.

One side of the brain was selected for analysis from each patient. Selection rule is demonstrated in Fig. 1.

This process resulted in separation of the selected OR’s into three groups:

1. OR’s with no discernable lesions;
2. OR’s with lesions partially crossing the tract;
3. OR’s with lesions occupying the entire cross-section of the tract.

**Fig 2:**
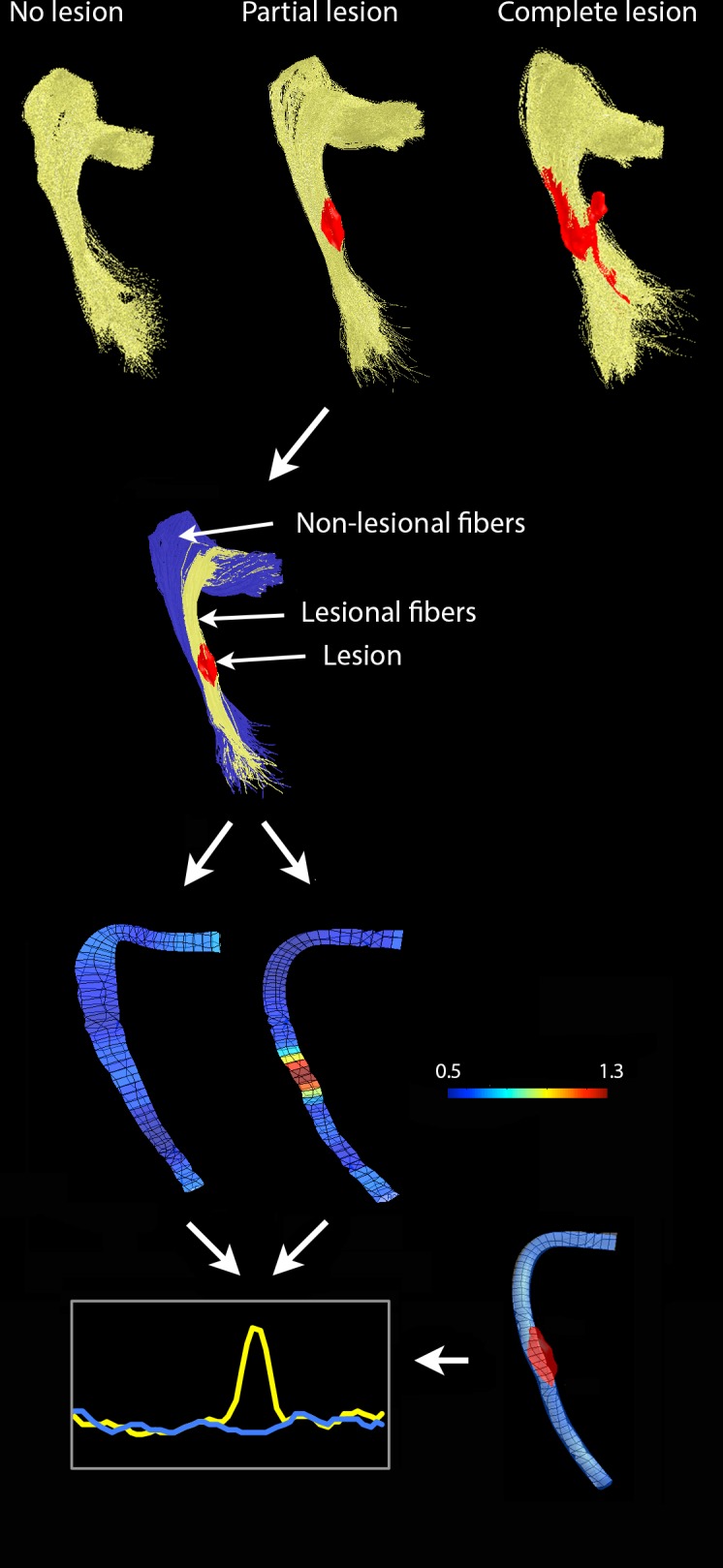
Segmentation and analysis of OR fibers. Top raw-Identification of 3 optic radiation groups. Second row-segmentation of OR in patients with partial lesions (red) into lesional (yellow) and non-lesional (blue) fibers. Third row-profile of diffusivity indices calculated for lesional and non-lesional fiber tructs based on 50 equally spaced nodes. RD profile is shown here as an example. Profile's width reflects the amount of spatial dispersion of the fibers. Colour scale represents the magnitude of RD (10^-3^ mm^2^/s). Bottom right-position of the lesion from co-registered T2 FLAIR image displayed on the 50-nodes template. Bottom left-asymmetry analysis of the diffusivity measures (lesional fibers-red, non-lesional fibers-blue). Position of the lesion indicated by grey box.

Where lesions partially crossed the OR, fibers were further sub-divided into fibers traversing the lesions (lesional fibers) and fibers with no discernable pathology throughout the entire length of the OR (non-lesional fibers) (Fig. 2).

### Calculation of diffusivity indices

Diffusivity indices including Fractional Anisotropy (FA), Mean Diffusivity (MD), Axial Diffusivity (AD) and Radial Diffusivity (RD) were calculated at multiple points along the OR for each subject individually by segmenting the entire length of the OR between the LGN and occipital cortex into 50 equally spaced nodes and taking a weighted average of the measurements of each individual fiber at that node (so called “fiber core” [15]). In patients with lesions partially involving the OR, diffusivity of lesional and non-lesional fibers was analysed separately (Fig. 1) and relative difference (asymmetry) of each diffusivity index between the two tracts was calculated at each node as follow:

(lesional fibres - non lesional fibres)/non-lesional fibres.

The exact position of each lesion on the individual 50-node template of lesional fibers was identified by co-registering to the OR lesion mask.

Due to spreading of the fibers of the posterior part of the OR along *sulcus calcarinus*, last five nodes were excluded from the analysis.

Averaged diffusivity indices for individual patients were also calculated for the entire tract.

### Statistics

Statistical analysis was performed using SPSS 21.0 (SPSS, Chicago, IL, USA). Pearson correlation coefficient was used for bivariate correlation. One-way ANOVA (Tukey post hoc test) was used to assess group differences between MS patients and healthy controls for average optic radiation DTI parameters. Student’s t-test was used to identify regional optic DTI abnormalities at each OR node. Linear Regression Model (backwards elimination) was performed to examine the effect of variable factors on diffusivity of OR NAWM. Univariate General Linear Model adjusted for age, sex and duration of the disease was used to analyse differences between multiple groups. Reported B values are Standardized coefficients.

## Results

Fifty RRMS and 15 healthy controls were enrolled. The OR on one side was analysed. No OR lesions at least on one side of the brain were detected in 24 patients. Of the remaining 26 patients, 21 had lesions partially involving the OR and 5 patients had lesions crossing the entire OR.

Demographic data are presented in Table 1. There was no significant difference between the three MS groups in terms of disease duration (one-way ANOVA, p>0.05). Age was also comparable between all groups (including healthy controls) (one-way ANOVA, p>0.05). However, since patients with completely lesioned OR were on average almost 10 years older compare to other groups, age was included as covariate in all statistical analysis.

**Table 1:** Demographic data.

	n	Age	F/M	ON history	Disease duration, y
Healthy controls	15	37.2+/-13.2	9/6	n/a	n/a
Patients without OR lesions	24	40.2+/-10	13/11	7	5.4+/-3.7
Patients with partially lesioned OR	21	37.0+/-12.5	16/5	11	4.0+/-2.3
Patients with completely lesioned OR	5	47.8+/-12.6	1/4	3	5.6+/-3.0

### Analysis of diffusivity in OR groups

Analysis of the entire patient cohort demonstrated significant difference from healthy controls for all diffusivity indices, which was particularly strong for RD. Group analysis revealed that this difference was driven by the patients with OR lesions. Thus, no difference between HC and patients without OR lesions was found for all diffusivity measures. In contrast, diffusivity measures of OR fibers in patients with OR lesions significantly differed from HC, especially in the patient group with completely lesioned OR. Namely, values of AD and RD were significantly elevated compared with the HC group. As a result, MD was also significantly increased relative to HC, while FA demonstrated significant reduction, possibly due to a greater increase in RD relative to AD (Table 2).

**Table 2:** Diffusivity measures in MS patients and healthy controls.

	AD	RD	FA	MD
Controls	1.32+/-0.05	0.57+/-0.03	0.5+/-0.03	0.82+/-0.03
All MS patients	1.37+/-0.09 p = 0.03*	0.62+/-0.07 0.002*	0.47+/-0.04 p = 0.04*	0.87+/-0.07 p = 0.003*
Patients without OR lesions	1.34+/-0.07 p = 0.8	0.59+/-0.05 p = 0.45	0.49+/-0.04 p = 0.7	0.84+/-0.05 p = 0.5
Patients with partially lesioned OR	1.43+/-0.09 p = <0.001*	0.67+/-0.06 p<0.001*	0.46+/-0.03 p = 0.001*	0.92+/-0.06 p = <0.001*
Patients with completely lesioned OR	1.50+/- 0.1 p = <0.001*	0.78+/-0.07 p = <0.001*	0.41+/-0.02 p = <0.001*	1.02+/-0.08 p = <0.001*

^a.^Student’s t-test was used to compare entire MS patient group with healthy controls, while one-way ANOVA (Tukey post hoc test) was used for comparison of individual groups (p represents statistical difference from healthy controls).

In concordance with previous studies of the OR [11], there was significant variation of all DTI indices along the tract including for healthy controls.

**Fig 3:**
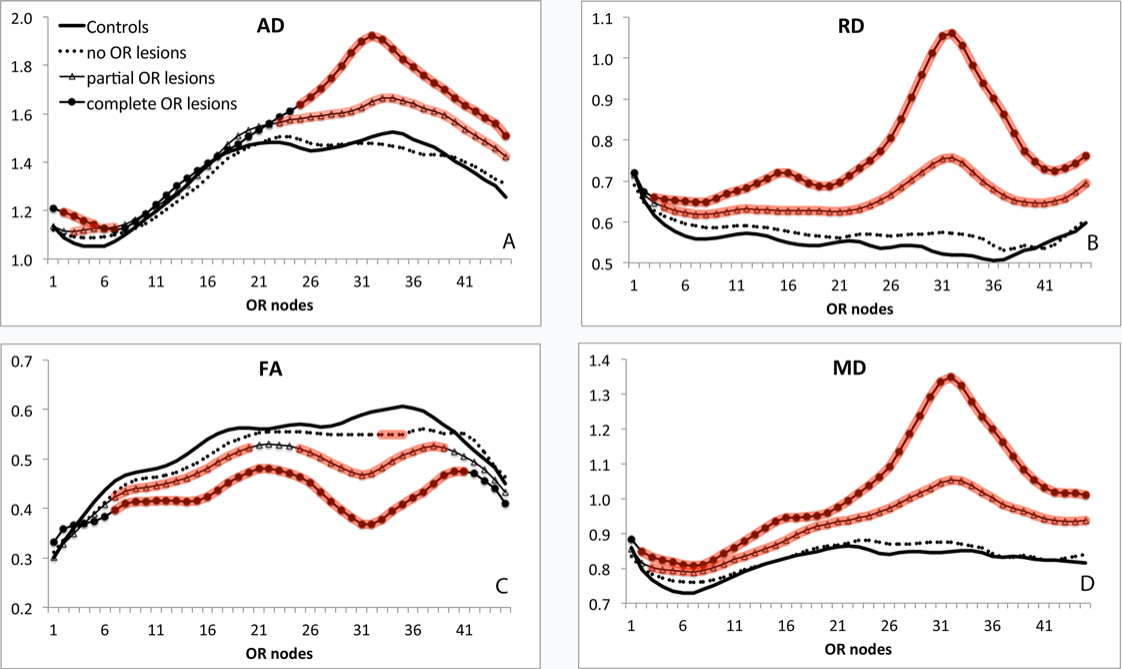
Profiles of OR diffusivity indices for three MS groups vs normal controls. Nodes with diffusivity values significantly different from corresponding points in healthy controls are highlighted by red (Student t-test). A. Axial Diffusivity. B. Radial Diffusivity. C. Fractional Anisotropy. D. Mean Diffusivity. Vertical scale-Units of diffusivity (except for FA): 10^-3^ mm^2^/s. Horizontal scale represents nodes from LGN to occipital cortex.

The tract profile of the diffusivity measures, presented in Fig. 3 revealed no difference between HC and patients without OR lesions for all diffusivity indices along the entire OR. In contrast, both groups of patients with OR lesions (partial and complete lesions) demonstrated significantly increased AD, RD and MD, but reduced FA (more prominent in a latter group). While changes in RD, MD and FA were particularly high in the third quarter of the OR, they were seen along the entire length of the tract. Increase in AD, however, was limited to the posterior half of the optic radiation.

### Analysis of non-lesional fibers

Acute lesions and following retrograde and Wallerian degeneration can cause significant diffusivity alteration[10] [11]. Therefore, in order to exclude the direct effect of acute inflammation, we examined non-lesional fibers separately.

In the OR without lesions all fibers were included in analysis (24 patients), while in the OR with partial lesions, only fibers not traversing the lesions were selected (21 patients).

While patients without OR lesions demonstrated similar value of RD as compared to healthy controls, there was a significant increase of the RD in non-lesional fibers of patients with partial OR lesions (Table 3).

**Table 3:** Average diffusivity indices for two groups of non-lesional OR fibers (*p* represents statistical difference from healthy controls).

	AD	RD	FA	MD
Healthy controls	1.32+/ 0.05	0.57+/-0.03	0.5+/-0.03	0.82+/-0.03
Fibers from OR with no lesions	1.34+/ 0.07 p = 0.6	0.59+/-0.05 p = 0.2	0.49+/-0.04 p = 0.6	0.84+/-0.05 p = 0.3
Non-lesional fibers from partially lesioned OR	1.37+/- 0.09 p = 0.07	0.63+/-0.04 p<0.001*	0.48+/-0.03 p = 0.01*	0.88+/-0.04 p<0.001*

**Fig 4:**
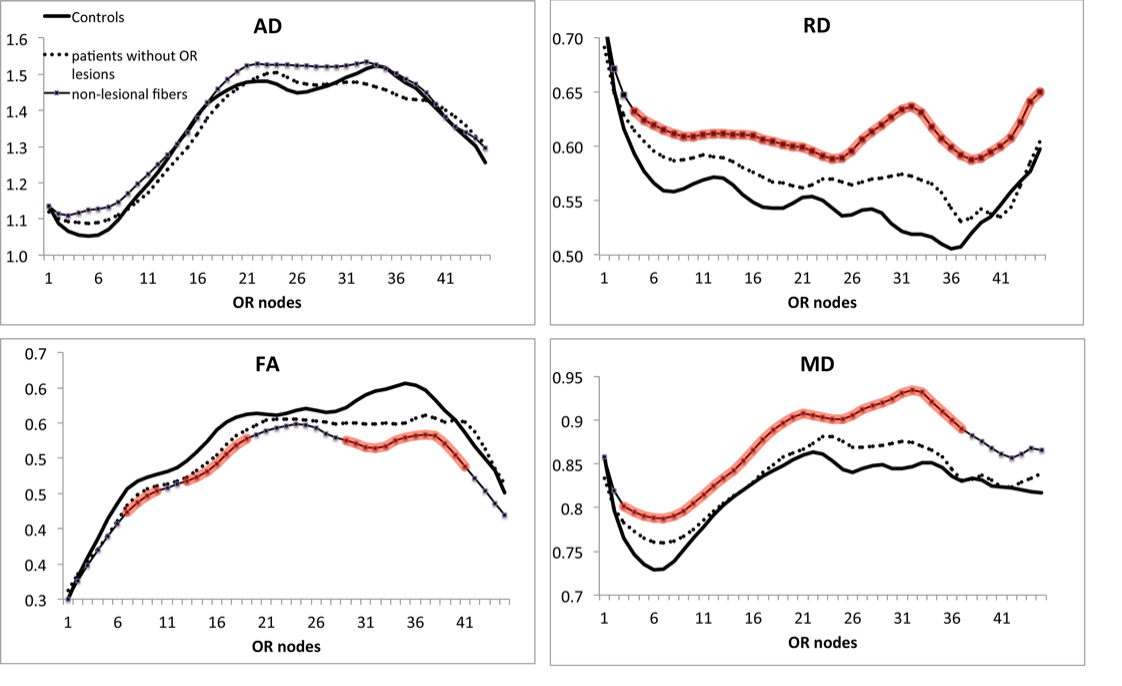
Profiles of OR diffusivity indices of patients without OR lesions and non-lesonal fibers of patients with partial lesions vs normal controls. Nodes with diffusivity values significantly different from corresponding points in healthy controls are highlighted by red (Student’s t-test). Units are the same as in Fig. 3.

This increase was observed along the entire length of the OR (Fig. 4). In contrast, AD showed no difference between the groups. The tract profile of AD also showed similar values for 3 groups along the entire OR.

As a consequence, FA and MD of the OR fibers in patients without OR lesions did not differ from those measured in HC. However, MD was significantly elevated, while FA was moderately reduced in non-lesional fibers of patients with partial OR lesions as compared to HC.

However, further analysis revealed that the total T2 lesion volume (T2LV) outside of the OR was three times greater in patients with OR lesions compared to those without (9953mm^3^ vs 2884mm^3^, p<0.001, Student’s t-test), suggesting the presence of more active disease in patients with OR lesions, which may potentially contribute to this apparent association.

Therefore a Univariate General Linear Model adjusted for T2LV outside of OR, was applied to analyse OR DTI metrics. Using this model, we found no difference between three groups (HC, patients without OR lesions and non-lesional fibers of patients with partial OR lesions) for all DTI indices (p = 0.2, p = 0.5, 0.4 and 0.2 for RD, AD, FA and MD respectively). This result suggests that the global lesion burden, rather than local OR lesions, is significantly associated with the RD of non-lesional OR fibers.

This hypothesis was further corroborated by the regression analysis where all 45 patients were analysed together. While it demonstrated significant correlations of RD (but not AD) with T2LV within and outside of the OR, the correlation was much stronger with T2LV outside of OR. Additionally, FA and MD correlated with T2LV outside of the OR, but not with OR T2LV (Table 4).

**Table 4:** Correlation between diffusivity indices and lesion volume within and outside of the OR.

	AD	RD	FA	MD
Lesion volume within the OR	0.11 p = 0.5	0.33 p = 0.025*	-0.28 p = 0.06	0.28 p = 0.06
Lesion volume outside of the OR	0.23 p = 0.1	0.54 p<0.001*	-0.35 p = 0.02*	0.46 p = 0.001*

A high correlation between T2LV within and outside of the OR has been noted previously [13][10] and confirmed in the current study cohort (r = 0.72, p<0.001). Therefore, to disentangle the effect of lesions inside and outside of the OR on diffusivity metrics, we employed a Linear Regression Model that, apart from lesion volume, also included disease duration, age, gender and a history of optic neuritis (ON).

The model explained 38% of the observed increase in RD in non-lesional fibers (p<0.001) and demonstrated a significant effect of T2LV outside of the OR (p = 0.001, b = 0.62) and, to a lesser extent, disease duration (p = 0.02, b = 0.31) and a history of ON (p = 0.03, b = 0.28), on RD. The model showed no significant associations of any of the variables with AD (p = 0.73). Accordingly, the model explained 23% of the observed FA reduction (p = 0.02) and 21% of the MD increase (p = 0.02). T2LV outside of the OR was the only variable with a significant effect on MD (p = 0.01, b = 0.54), while disease duration (p = 0.03, b = -0.32) and history of ON (p = 0.02, b = -0.33) significantly impacted FA.

**Fig 5:**
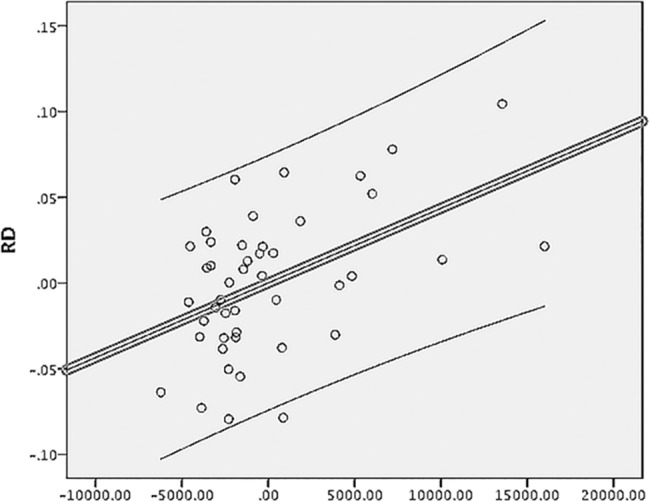
Partial regressions plot of RD of the non-lesional OR fibers vs T2LV outside of the OR. Axes represent residuals. Linear fit and 95% individual confidence intervals are shown.

Excluding cases that had a history of ON increased the predictive power of the model for both RD and MD even further (60%, p<0.001 and 48%, p = 0.006 for RD and MD respectively) (Fig. 5). Only T2LV outside of the OR retained significance for both metrics (p<0.001, b = 0.76 and p = 0.003, b = 0.63 for RD and MD respectively). The model for FA lost significance (p = 0.06), and remained non-significant for AD (p = 0.3).

Since ON may potentially affect diffusivity metrics (and result in increase of RD) we also grouped patients based on a history of ON and analysed diffusivity indices of non-lesional fibers in each group. Analysis revealed no difference between groups for all diffusivity metrics (p>0.05 for all indices).

The result of the modeling, therefore, emphasizes strong association between increase of RD in non-lesional fibers of the OR and T2LV outside of the OR.

### Lesional vs non-lesional fibers

A significant proportion of patients in the study cohort (42%) presented with lesions partially crossing the OR. There was a significant increase in the average RD (0.63+/-0.04 vs 0.70+/-0.05), AD (1.37+/- 0.09 vs 1.44+/- 0.09) and MD (0.88+/-0.04 vs 0.95+/-0.06) and a reduction in the average FA (0.48+/-0.03 vs 0.44+/-0.03) in lesional fibers compared with non-lesional fibers (p<0.001 for all, paired t-test).

Since the OR consists of tightly packed parallel fibers, it is reasonable to assume that, in the healthy brain, the diffusivity of all fibers at particular cross-section of the OR is similar. Consequently, a potential difference in diffusivity between corresponding nodes of lesional and non-lesional fibers of the same OR is likely to be a result of tissue restructuring caused by local pathological changes. Therefore, the difference in diffusivity indices between lesional and non-lesional fibers was calculated at each node along the OR in order to examine diffusivity changes produced by lesions and to evaluate the potential effect of Wallerian and retrograde degeneration on diffusion properties of the OR fibers.

**Fig 6:**
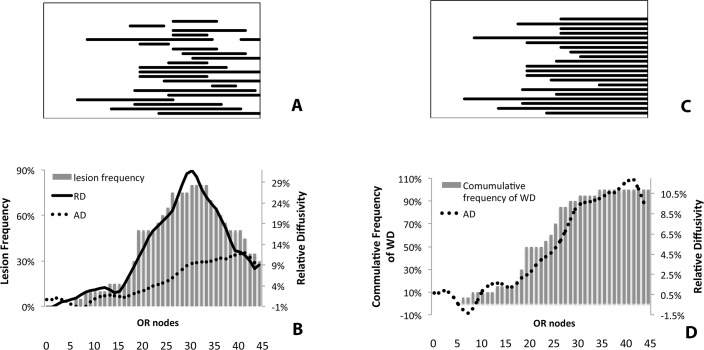
Distribution of RD, AD, T2 FLAIR lesions and the fibers affected by Wallerian degeneration along the OR in patients with partially lesioned OR. A. Position of individual OR lesions between LGN (on the left) and occipital cortex (on the right). B. Profiles of averaged ΔRD and ΔAD superimposed on histogram of lesion frequency along the OR. The ΔRD profile mirrors the distribution of lesions along the OR, while ΔAD slowly increases toward the distal pole of the OR. C. Hypothetical distribution of the fibers affected by Wallerian degeneration along the OR. D. Averaged ΔAD profile superimposed on histogram of the fibers affected by Wallerian degeneration.

The position of lesions along the OR was identified using the template described above in all 21 patient with partial OR lesions. The highest concentration of lesions was observed in the third quarter of the OR, while no lesions were identified in the part of the OR in close proximity to LGN (Fig. 6A-B).

A relative increase in RD in lesional vs non-lesional fibers (ΔRD) was maximally observed in the third quarter of the OR, where it reached 30% (Fig. 6B, solid line). Remarkably, the distribution of ΔRD along the OR mirrored the distribution of OR lesion frequency (Fig. 6A). This relationship was substantiated by a high topographical correlation between the frequency of lesions along the OR and ΔRD (r^2^ = 0.95, p<0.001).

**Fig 7:**
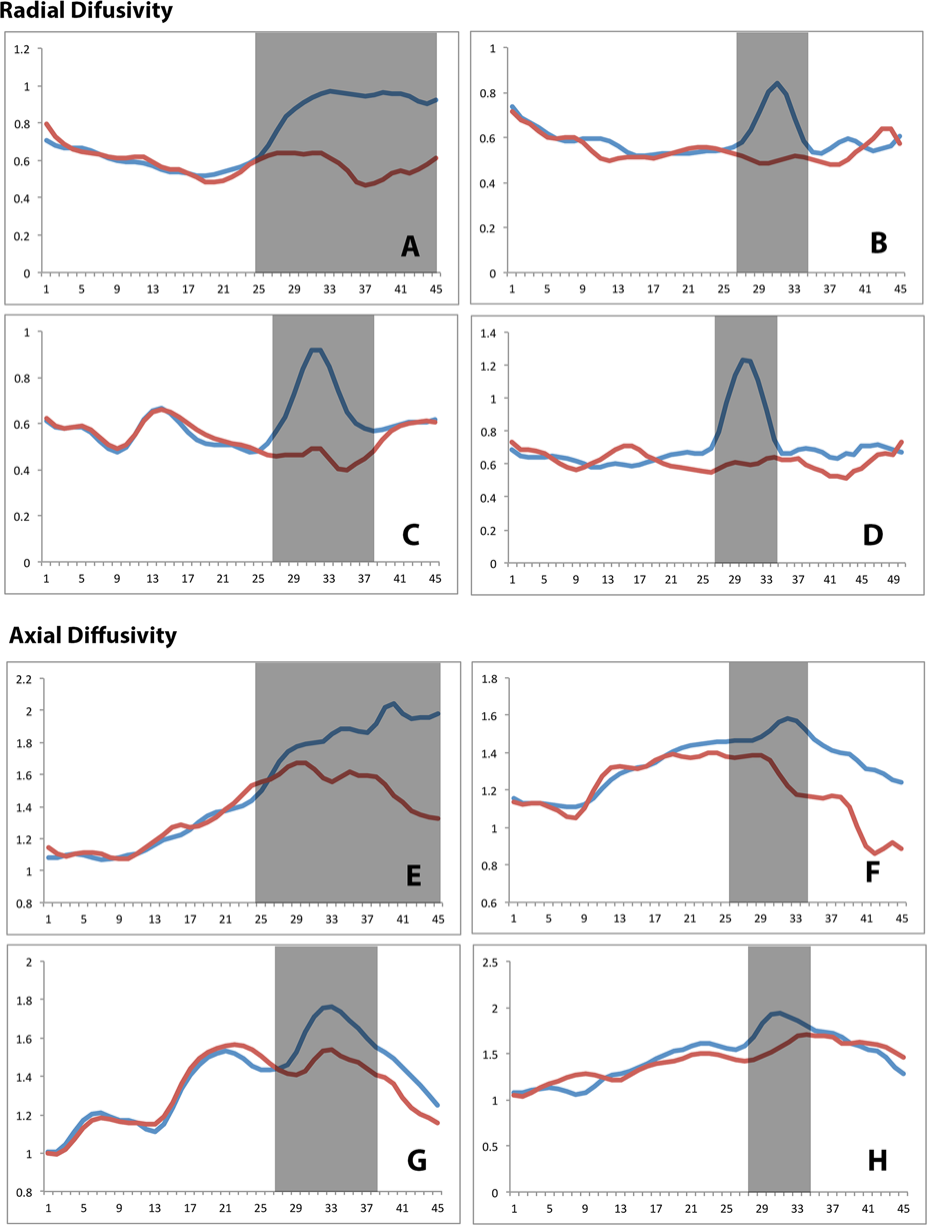
Examples of RD and AD profiles along the OR. A-D Examples of lesional (red) and non-lesional (blue) RD profiles of the OR.E-H Examples of lesional (red) and non-lesional (blue) AD profiles of the OR. Position of the lesion indicated by grey box.

Moreover, the examination of RD of individual cases revealed that the ΔRD was not only substantially elevated within the lesions, but this difference was strictly limited by the lesional border, whilst outside the lesions (both proximally and distally) RD between the two fiber groups was similar (Fig. 7A-D).

In contrast to RD, AD displayed a different pattern of results. Firstly, the magnitude of the relative increase of AD in the lesional fibers (compared with non-lesional fibers) (ΔAD) was smaller compared to changes of RD (12% maximum). Also, the distribution of the ΔAD along the OR differed from the distribution of the ΔRD and did not match the pattern of lesional distribution. Instead, averaged ΔAD gradually increased along the anterior-posterior course of the OR with a tendency to plateau at the occipital pole (Fig. 6B, dotted line).

Analysis of individual cases revealed that, while there was no ΔAD increase in the proximal (in relation to lesion) part of the OR, in majority of patients the elevation of AD in lesional fibers was not limited by the distal border of the lesion, but continue all the way to occipital cortex (Fig. 7E-G). Few patients, however, showed minimal, if any, AD changes between lesional and non-lesional fibers (Fig. 7H).

**Fig 8:**
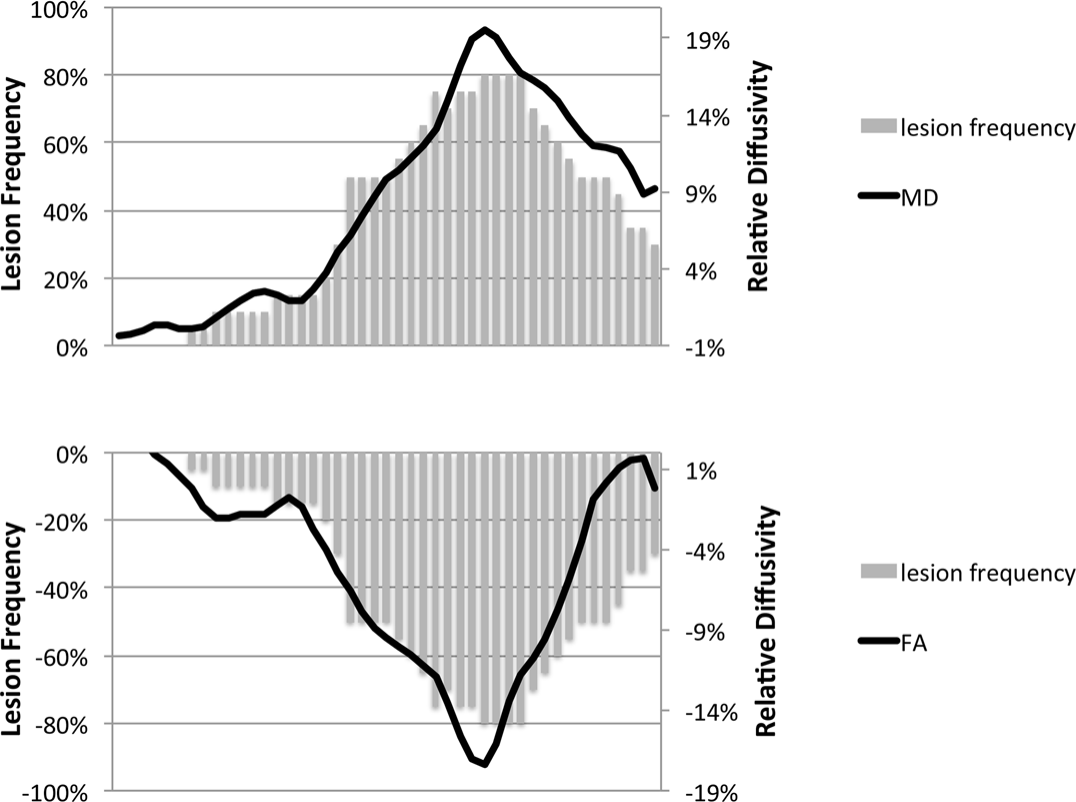
The profiles of MD and FA along the OR superimposed on the histogram of lesion frequency.

The relative alterations in RD and AD resulted in predictable changes in FA and MD. Thus, the MD of the lesional fibers was significantly elevated in middle portion of the OR, which corresponded to the region which had the highest prevalence of the OR lesions, but still remained high in the posterior part of the OR compared with non-lesional fibers. Conversely, FA demonstrated a sharp decline in the middle part of the OR, normalizing at its proximal and distal extent (Fig. 8).

## Discussion

Elucidation of the biological substrates for altered diffusivity in MS lesional and NAWM tissue will critically inform disease pathogenesis *in-vivo* and facilitate the development of robust biomarkers of axonal and myelin integrity that can be incorporated into clinical trials. However, since white matter diffusivity is highly variable, even along the single tract, diffusion coefficients within the lesions must be compared to normal tissue in corresponding (anatomically matched) regions of the brain [16][17], which is not always technically or anatomically feasible. On the other hand, NAWM in MS is heterogeneous, containing axons that are variably oriented and undergoing different stages of degeneration as a result of transection in distant lesions, which may have different, and potentially opposing, effects on diffusivity [18]

In the current study we carefully segmented fibers of a single tract into separate groups based on their relationship to lesions. We then calculated diffusivity measures in predefined regions along the white matter tracts and compared the diffusivity metrics with the corresponding segments of the non-lesional fibers of the same tract. This approach, which allowed us to use the fibers unaffected by lesions as an “internal control”, is only possible to employ in well-defined, long and reasonably wide fiber tracts, that are not “contaminated” by crossing fibers [18].

The OR was chosen to undertake this analysis for several reasons. Firstly, advances in tractography permit reliable separation of the OR from the surrounding cerebral white matter. Secondly, axons of the LGN neurons, which form the OR, are coherently oriented, facilitating accurate measurement of relative diffusivity change along axonal bundles in the tract. Thirdly, the OR does not contain a significant number of crossing fibers, which can potentially (and sometimes paradoxically) alter diffusivity [15][19]. This point is especially pertinent considering the issues that surround misalignment between corresponding eigenvectors with the underlying tissue structures [9]. Finally, the OR is a frequent site of MS lesions and, as a consequence, is likely to be subjected to retrograde and Wallerian degeneration.

Fibers that form the OR are spread relatively widely, and as such MS lesions often only cross part of the tract, leaving some fibers completely lesion-free along the entire length of the OR. This presents a unique opportunity to directly compare the diffusivity of lesion-traversing fibers with corresponding non-lesional fibers. This “asymmetry analysis” is highly sensitive to subtle localised DTI changes since it takes into account both inter- and intra (i.e. along the OR tract)-subject diffusion variability.

### Diffusivity in non-segmented OR

We corroborated previous studies [10][11] that have reported significantly increased RD, AD, MD and reduction in FA in the OR of MS patients. Our analyses of diffusivity in un-segmented OR demonstrate that progressive worsening of all DTI indices is related to the degree of the OR T2 lesion burden. This observation confirms that MS lesions significantly contribute to DTI abnormalities, and is consistent with previously reported diffusion abnormalities in OR lesions relative to NAWM [11].

While a relatively evenly distributed patterns of reduced FA and increased MD, RD and AD along the OR has been previously reported [10] [11], our data suggest that diffusivity is predominantly altered in the central and posterior parts of the OR. This discrepancy could potentially relate to differential patterns of lesion distribution between study cohorts.

### Diffusivity in lesional vs non-lesional fibers in patients with partial OR lesions

Acute inflammatory demyelination results in the transection of a significant number of axons within the MS lesion [20]. As a result, both the proximal and distal parts of lesioned fibers undergo degeneration. Since MS lesions typically do not encompass the entire cross-section of the OR, fibers free of the lesions were used as an internal control to examine the effect of tissue remodeling caused by lesions and lesion-induced axonal degeneration on OR diffusivity.

Asymmetry analysis showed a substantial increase in RD within the lesional fiber track, which was topographically constrained by the T2-visible lesion(s). Interestingly, RD measured proximal and distal to the lesion was similar in corresponding nodes of lesional and non-lesional fibers, potentially indicating poor sensitivity of this metric for axonal degeneration. Group asymmetry analysis revealed that the distribution of the lesions along the OR mirrored the relative RD increase in the lesional fibers, with correlation between the two groups exceeding 98%.

Axial diffusivity in lesional fibers was also significantly increased, albeit to a lesser degree than RD. Similar to RD, AD within the proximal OR did not differ significantly between lesional and non-lesional fibers. However, elevation of the ΔAD did not correspond with topographic lesion frequency and was not limited to the site of the lesion, but increased gradually toward the visual cortex, plateauing at the end, a trend that was particularly visible in the averaged data.

This discrepancy in distribution between ΔRD and ΔAD implies that different pathophysiologic processes may underlie the observed changes of AD and RD of the lesional OR fibers.

Why, therefore, are lesions so uniquely susceptible to increased perpendicular diffusivity, and what is the pathological substrate for the characteristic alteration of AD in lesional fibers described above?

Let us consider lesions first. Chronic MS lesions are characterized by demyelinated axons, extensive gliosis and axonal loss [21]. Numerous axons are transected in the acute inflammatory lesion and subsequently degenerate. Since tissue restructuring caused by axonal loss extends far beyond the lesion in the form of Wallerian and retrograde degeneration, axonal loss is unlikely to be the principal substrate for diffusion changes restricted to the lesion.

However, axons, which survived the initial inflammatory attack, are completely denuded of myelin within the lesion (with the possible exception of the plaque margin) [22]. In some lesions demyelinated axons are separated from each other by fibrous astroglial processes, whilst in other lesions the nerve fibers are only separated by a normal or widened extracellular space [23][24][20].

While pathological expansion of extracellular space spreads beyond the lesion into NAWM [21][25], the degree of myelination abruptly changes at the lesion border. Therefore, loss of myelin and its partial replacement with glia represents the major difference between intra-lesional and extra-lesional components of the same fiber track.

The space vacated by elimination of myelin can completely collapse (i.e. taken by neighboring demyelinated axons), filled with extracellular space and/or be re-populated by glial cells [21]. However, since demyelinated axons and glial cells have only single-layer membrane and, therefore, display similar (and relatively fast) water molecules exchange rates, all potential outcomes are likely to have similar effect on RD [26]. Therefore, our findings strongly suggest that lesion-confined relative increase in RD is directly related to the loss of myelin. This concurs with previous reports demonstrating significant association of increased RD with degree of lesional demyelination (see [27] for most recent review).

The nature of altered AD in MS, which has been frequently attributed to axonal loss [28], is critically informed by an understanding of the mechanisms of axonal degeneration. Axonal degeneration following axonal transection within the acute inflammatory MS lesion progresses in both directions. However, the molecular mechanisms and morphological restructuring in axons proximal and distal to the site of transection, which lead to retrograde and anterograde (Wallerian) degeneration respectively, are different [29][30][31][32].

Apoptosis is the intracellular suicide program. It can be activated by neuroaxonal damage during adult life [32] and typically results in fragmentation and complete elimination (via phagocytosis) of entire neuron, including the neuronal body, axon and surrounding myelin, leading to tissue collapse [33][34]. In the case of axonal transection, only the neuronal cell body and proximal portion of the axon and its myelin sheath are subjected to apoptotic destruction [35][36]. While the role of neuro-axonal apoptosis in MS is not well understood [37][38], convincing proof of apoptosis has been demonstrated in animal models of the disease [39][40][41].

There is also strong evidence suggesting that, at least in the visual pathway, degeneration of the neuronal body and the attached portion of the transected axon is typically mediated by apoptosis [42][43][44][41][45][46][47][48].

LGN neurons, which project exclusively to the visual cortex, may be particularly prone to apoptotic cell death due to complete target deprivation subsequent to axonal transection [46][34]. The proximal component of transected LGN axons, therefore, is likely to be completely eliminated by macrophages following apoptosis of their cell bodies, and vacated space taken by preserved neighboring axons [35]. While apoptosis of LGN neurons has not been demonstrated in humans (potentially reflecting the rapid kinetics of apoptosis signaling cascades) [49], the elimination of RGC neurons (which is closest neighbor to LGN neurons) and their axons following optic neuritis has been successfully measured by Optical Coherence Tomography (see [50] for review).

Following axonal transection, the latency of proximal axonal degeneration (that follows LGN apoptosis) may be significantly longer than degeneration of distal axons [51][52]. This may explain the frequent observation of terminal axonal spheroids in MS lesions, a hallmark of disrupted axonal transport in transected, but still surviving, proximal axons [53][54].

The mechanism of the Wallerian degeneration differs significantly. The part of the axon distal to the injury site displays rapid and widespread breakdown of the axonal cytoskeleton, destruction of internal organelles, and ultimately granular disintegration [55]. The entire process takes several days and is similar in both peripheral and central nervous systems. In the periphery, it is followed by rapid degradation of myelin (due to massive infiltration of macrophages and proliferation of Schwann cells) and finally, by fibrosis and atrophy of the affected fiber tracts. Conversely, in the CNS the myelin sheath remains relatively intact for long periods, possibly years [31][56][57][58]. Delayed degradation and removal of myelin in CNS has been attributed to lack of macrophages in degenerating distal tracts and difference in glial reaction [57].

Preserved myelin sheaths of the distal portion of degenerated axons initially form empty tubes or arrays of myelin ovoids [59][60], which are gradually replaced by glial tissue and loosely arranged extracellular space [61][62]

**Fig 9:**
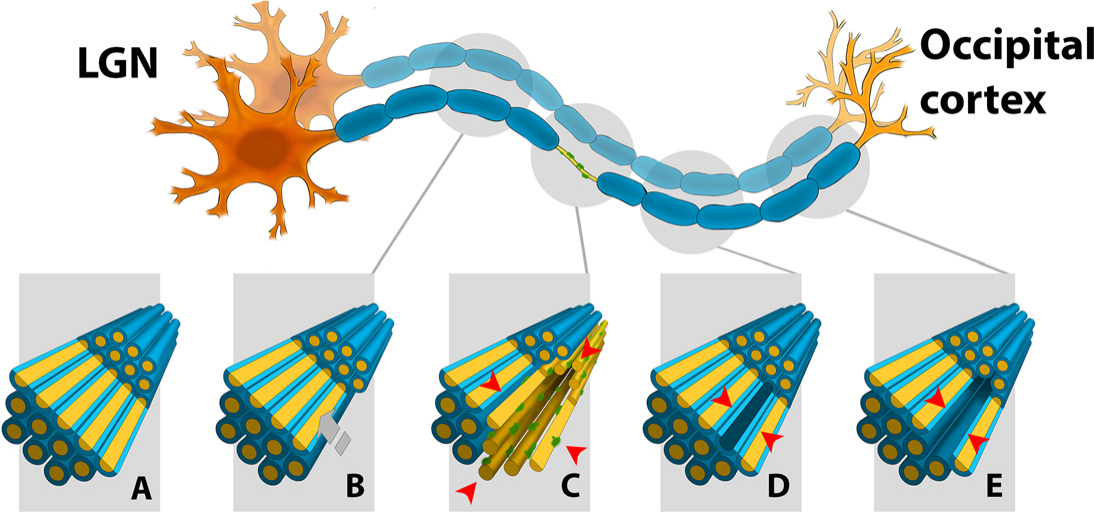
Schematic representation of the outcome of the partial OR lesion. A. normal structure of the OR: axons-yellow, myelin sheaths-blue, B. proximal (to the lesion) part of OR: space occupied by the apoptotically removed axon is replaced by neighbouring axons and the tissue shrinks, C. site of the partial lesion (indicated by red arrows): lesional part (bottom right) represented by demyelinated (naked) axons and glial cells (green), which surround the empty space left by the degenerated axon, non-lesional part (top left) represented by normal (myelinated) axons, D. distal (to the lesion) part of OR: an early stage-empty myelin tube (indicated by red arrows) left by degenerated axon is surrounded by intact axons, E. distal (to the lesion) part of OR: a late stage-empty “cylinder” of extracellular matrix (indicated by red arrows) left by degenerated axon is surrounded by intact axons.

It is reasonable to assume that the extracellular space, which forms as a result of this process, will follow the topographic profile of degenerated axons and empty myelin tubes. In the OR, where tightly packed long axons run in parallel, this would result in tiny “cylinders” of empty myelin tubes or extracellular matrix (depending on the chronicity of the lesion) dispersed between normal axons, extending longitudinally from the site of lesional transection to the posterior pole of the OR. As only a small percentage of OR neurons die as a result of a single attack [25], it is likely that each “cylinder” is surrounded by preserved, but demyelinated axons within the lesion, and their myelinated continuation distal to the lesion (Fig. 9).

If we extrapolate from this pathological model, it would not be expected to find an alteration of RD and AD proximal to the site of axonal transection, since after elimination of apoptotic neurons the entire tract, while potentially shrunken, would consist of normal axons (Fig. 9B). Accordingly, our data clearly show no change in diffusivity of lesional fibers compared with non-lesional fibers in the proximal part of the OR.

Diffusivity of the distal part of the OR, however, is likely to undergo changes. RD within empty myelin tubes may remain restricted by preserved myelin sheaths (and, therefore, unaltered) (Fig. 9D). At the later time, when myelin remnants are finally cleared, perpendicular diffusion of water within the tissue may still remain restricted by the myelin lamellae of neighbouring intact axons (Fig. 9E). However, since the normal structure of the axoplasm is completely replaced by low density extracellular matrix (which is likely to have a higher rate of water mobility due to loss of organelles [2]), AD is expected to increase. Since Wallerian degeneration affects the entire distal portion of transected axons (Fig. 6C), the increase in AD should not be limited to the lesional site, rather continue along the OR all the way toward the visual cortex. Correspondingly, our data demonstrates this pattern of relative AD increase in lesional fibers in the majority of patients with partial OR lesions.

The cumulative volume of the “cylinders” (or, in other words, volume of WD) for the entire group is also expected to progressively increase towards the visual cortex, plateauing after the most distally placed lesion (Fig. 6D). Remarkably, the cumulative histogram of WD based on this distribution was tightly associated with the relative increase of AD in lesional fibers of the OR (r^2^ = 0.94, p<0.001).

It appears likely, therefore, that the relative increase in AD in the distal part of lesional fibers is largely related to the extent of axonal transection within the lesions and the subsequent distal axonal loss due to WD.

In addition, no change of AD has been found in non-lesional fibers of the OR, lending further support to the affiliation of AD with tissue restructuring caused by lesional axonal damage.

Recent publications suggest time-dependent character of AD alteration caused by axonal transection and following WD. Reduced AD is typically seen in the early (acute) stages of WD and has been ascribed to fragmentation of axons, which creates barriers to the longitudinal displacement of water molecules[63][64][27][28] [65] [66][7][67][68][69]. However, in chronically degenerated white matter AD tends to be elevated, a finding that has been attributed to axonal loss [70][71][72][73][63][27]. Moreover, longitudinal studies investigating diffusivity changes in fibers undergoing WD also showed that following initial drop, AD tends to recover [74][75], sometimes even above normal value [76]. These observations are consistent with our findings since none of the study patients demonstrated acute Gd-enhancing lesions at the time of the examination suggesting chronic pathology.

### Diffusivity in non-lesional OR fibers

Tissue damage in NAWM has been predominantly attributed to axonal loss resulting from WD [77][78]. The possibility of a primary inflammatory process associated with myelin loss has also been suggested (see [79] for review) and is certainly supported by the evidence suggesting the presence of microscopic lesions using 7T scans [80] [81]. However, *en masse* evaluation of all axons within NAWM region or fiber tract makes it impossible to separate the effect of various tissue pathologies on DTI [82].

OR tractography facilitates segmentation of the entire length of LGN axons, providing a unique opportunity to study NAWM fibers that do not traverse lesions, in isolation. By doing so, the potential effects of WD can be completely eliminated. Using this approach, we demonstrated a significant increase in RD in non-lesional fibers of NAWM (albeit on a lesser scale than seen within lesions). AD, on the other hand, was not altered compared with normal controls. This is consistent with a recent *in vivo* report demonstrating that spinal cord tracts devoid of lesions at any level may have abnormally increased RD [8].

In our study, the increase in RD in non-lesional NAWM fibers was proportional to the T2LV outside, but not within, the OR. The strong relationship with remote, but not local, T2 pathology implies that RD in non-lesional NAWM fibers is related to the overall burden of brain inflammatory activity rather than locally induced degenerative changes. While altered diffusivity in non-lesional fibers do not exclude the possibility of trans-synaptic changes in NAWM as a consequence of distant lesions, unilateral increase of RD (with no detectable alteration of AD) suggests potential involvement of demyelination, which is in agreement with recently published post-mortem study of MS brains [83]. This is also supported by the group analysis based on a history of ON, which revealed no difference between groups for all diffusivity metrics.

As RD in non-lesional OR fibres was relatively increased in patients with OR lesions, a partial volume effect from adjacent lesions cannot be excluded. However, since altered RD was observed along the entire OR, and was largely accounted for by T2LV outside of the OR, any partial volume effect is likely to be small.

### Utility of Fractional Anisotropy and Mean Diffusivity

MD and FA are composite indices derived from primary eigenvalues and were designed to describe the total amount of diffusion and relative degree of anisotropy. While the assessment of anisotropy is useful, it represents a simplified expression of water diffusion in a tissue [27]. Our study demonstrated that FA and MD do not add significantly to the information provided by eigenvalues. Furthermore, being dependent on combination of magnitude and polarity of eigenvalues, they may sometimes be misleading and should be interpreted with caution.

This is evidenced by the different relative distribution of AD and RD in lesional fibers (Fig. 8), leading to both measures becoming equal in posterior part of the OR, where similar, but moderately elevated values of both indices resulted in normalization of FA (since AD and RD offset one another), but increased MD. As a result the FA profile may potentially “under-estimate” lesional changes, while the distribution of MD may “over-estimate” it.

### Limitations of the study

Conclusions regarding the relationship between specific MS-related pathologies and diffusivity alterations are necessarily limited by the cross-sectional nature of this work. We believe that longitudinal studies of the OR in MS will determine whether a causative relationship can be established.

Manual ‘cleaning’ of OR fibers, a necessary element of probabilistic tractography, may also introduce some uncertainty regarding true size and position of OR. Extreme care, however, was taken not to remove fibers that follow the well-described trajectory of the OR. The fact that all ROIs were place manually constitutes another limitation of the study.

T1 hypointense lesions, which more robustly indicate severe tissue damage than T2 FLAIR hyperintense lesions, were not analysed in this study. Furthermore a comparison of these findings with a progressive MS cohort, in whom active inflammatory lesions are unlikely to develop, would potentially strengthen this work.

Finally, the relatively small sample size included in the current work prevented us from exploring a potential effect of disease-modifying therapies on the metrics studied. However, the measurement of asymmetry between lesional and non-lesional fibres within the same subject would dilute these potential effects.

## Conclusion

This study demonstrates the presence of tract-specific patterns of altered diffusivity in MS, providing further evidence that DTI is a sensitive marker of tissue damage in both lesions and NAWM. Our results suggest that, at least within the OR, parallel and perpendicular diffusivities are affected by tissue restructuring, related to distinct pathological processes.

A marked increase in RD was topographically linked with visible focal T2 lesions and potentially relates to the degree of lesional myelin loss. A relative elevation of AD in lesional fibers was observed in a distribution consistent with WD, while diffusivity in the proximal portion of transected axons was normal.

A moderate, but significant elevation of RD in OR non-lesional fibers was strongly associated with the global T2 lesion burden and is probably related to microscopic demyelination undetected by conventional MRI.

This work highlights the utility of the visual system in elucidating the pathological substrates for altered diffusion metrics in MS. Future longitudinal studies will determine the robustness of our findings and the applicability of OR DTI as a biomarker of axonal and myelin integrity for clinical trials of emerging pro-reparative therapies in MS.
